# Improving Mood and Cognitive Symptoms in Huntington's Disease With Cariprazine Treatment

**DOI:** 10.3389/fpsyt.2021.825532

**Published:** 2022-02-10

**Authors:** Maria Judit Molnar, Viktor Molnar, Mariann Fedor, Reka Csehi, Karoly Acsai, Beata Borsos, Zoltan Grosz

**Affiliations:** ^1^Institute of Genomic Medicine and Rare Disorders, Semmelweis University Budapest, Budapest, Hungary; ^2^Global Medical Division, Richter Gedeon Plc., Budapest, Hungary

**Keywords:** Huntington's disease, cariprazine, apathy, cognitive decline, behavioral alteration, mood

## Abstract

In Huntington's disease (HD), the main clinical symptoms include depression, apathy, cognitive deficits, motor deficiencies and involuntary movements. Cognitive, mood and behavioral changes may precede motor symptoms by up to 15 years. The treatment of these diverse symptoms is challenging. Tetrabenazine and deutetrabenazine are the only medications specifically approved for Huntington's chorea, but they do not affect the non-motor symptoms. For these, antidepressants, antipsychotics, and benzodiazepines have demonstrated benefit in some cases and can be used off-label. These drugs, due to sedative side effects, may negatively influence cognition. Sixteen patients having HD received a 12-week off-label cariprazine (CAR) treatment (1.5–3 mg/day). Cognitive performance and behavioral changes were measured by the Addenbrooke Cognitive Examination (ACE) test, the Cognitive and Behavioral part of the Unified Huntington's Disease Rating Scale (UHDRS), and the Beck Depression Inventory (BDI). Mixed model for repeated measures was fitted to the data, with terms of visit, baseline (BL) and their interaction. Cariprazine treatment resulted in the following changes from BL to week 12, respectively: the mean score of BDI decreased from 17.7 ± 10.7 to 10.0 ± 10.7 (*p* <0.0097), while the Behavioral Assessment score of the UHDRS decreased from 54.9 ± 11.3 to 32.5 ± 15.4 (*p* < 0.0001); ACE score increased from 75.1 ± 11.0 to 89.0 ± 9.3 (*p* < 0.0001); Cognitive Verbal Fluency score from 6.2 ± 2.5 to 7.7 ± 2.7 (*p* < 0.0103); Symbol Digit Test from 9.2 ± 6.9 to 12.3 ± 8.9 (*p* < 0.0009). Mild akathisia was the most frequent side effect, presenting in 2 out of 16 patients (12.5%). We conclude that CAR had a positive effect on depressive mood, apathy and cognitive functions in patients with early stage of HD. Based on the neurobiological basis of these symptoms, CAR can improve the dopamine imbalance of the prefrontal cortex. This draws attention to the transdiagnostic approach which supports the further understanding of the similar symptomatology of different neuropsychiatric disorders and helps to identify new indications of pharmaceutical compounds.

## Introduction

### Huntington's Disease

Huntington's disease (HD) is an autosomal dominantly inherited polyglutamate repeat expansion disease causing neurodegeneration in the brain. In the huntingtin (*HTT*) gene the expansion of an unstable polymorphic trinucleotide repeat (CAG) region located within the open reading frame at the 5′ end of the first exon is responsible for the disease. In HD individuals the range of the expanded CAG repeats is between 36 and 250 (1). There is an inverse correlation between the number of repeats with onset, severity and progression of the disease. However, at least 6 genes are known to have a modulating effect on disease manifestation (2). The pathomechanism is related to the CAG repeat expansion in the *HTT* gene, which results in complex pathophysiological changes (3) affecting mitochondrial function, mitophagy and immune system as well (4). The clinical picture is dominated by motor symptoms (chorea, at end stage akinetic-rigorous hypokinesis), and non-motor features, such as cognitive dysfunction (including executive dysfunction, planning difficulties, cognitive decline), depression, apathy, irritability and behavioral disinhibition (e.g., making inappropriate comments, impulsivity, hypersexuality). Non-motor symptoms can appear before the motor symptoms, and are very strong predictors of loss of independence and quality of life.

### Role of Dopamine in Huntington's Disease

Dopamine (DA) as a major neurotransmitter has essential roles regulating motor function, motivation, reward/pleasure, spatial memory function, lactation, and nausea (5). Five subtypes of dopamine receptors are known and classified into two receptor classes, class D1 and class D2. D1 and D5 receptor subtypes belong to class D1, while D2, D3, and D4 subtypes belong to class D2. The two most important dopamine receptors in the pathophysiology of neuropsychiatric disorders are the D2 and D3. The highest expression of D3 receptors is localized in the islands of Calleja, but is expressed throughout the limbic circuits, including the prefrontal cortex (PFC) (6), while the highest expression of D2 is linked to the striatum. Three major dopaminergic pathways are thought to be involved in HD: the mesolimbic pathway, projecting from the ventral tegmental area to the ventral striatum in the forebrain; the mesocortical pathway projecting from the ventral tegmental area to the prefrontal cortex; and the nigrostriatal pathway connecting the substantia nigra and the caudate and putamen. These loops maintain physiological regulation on behavior and voluntary movement.

In HD, the dopamine balance in the striatum and the frontal lobe is altered, leading to changes in motion, cognitive and behavioral performance. In early stages of the disease, the amount of DA is increased while the expression of DA receptors is decreased. In later stages, similar to Parkinson's disease, the amount of DA declines (7, 8). First over- then under-production of DA mirrors the biphasic changes in motor symptoms characteristic of HD patients throughout the disease course (9, 10). Optimal function of the non-motor symptoms depends on the constant level of DA. Both low and high levels of DA lead to behavioral, mood, and cognitive malfunction (11). Increasing evidence suggests the crucial role of the dopaminergic system in the development of HD symptoms, therefore DA-release modulating compounds might be a promising therapeutic option. DA stabilizing compounds, such as dopamine partial agonists, can increase or decrease DA receptor activity depending on the dopamine levels at the synapse.

### Treatment Approaches of HD

There is a definite unmet need for causative therapies in HD. Several approaches have been designed to reduce mutant huntingtin (mHTT) concentrations in the CNS such as (1) non-allele-selective antisense oligonucleotides (ASOs); (2) gene editing strategies, including zinc finger nucleases, transcription activator-like effector nucleases, clustered regularly interspaced short palindromic repeats (CRISPR-Cas 9) techniques; (3) gene therapy, and (4) stem cells reprogramming with single-stranded RNAs, mismatch-containing RNAs, antisense oligonucleotide, and small hairpin RNA (12). However, ultimate treatment solutions are not yet available, therefore the treatment of HD still heavily relies on symptomatic treatment.

Tetrabenazine (TBZ) and deutetrabenazine (deuTBZ) are approved for the treatment of motor symptoms in HD, such as chorea. TBZ is an inhibitor of the vesicular monoamine transporter 2 and its most prevalent dose-limiting side effects include somnolence, insomnia, depressed mood, akathisia, and parkinsonism (13). The deuterated form of hydrogen molecules in deuTBZ has a longer half-life requiring less frequent daily dosing, and likely having a better tolerability profile than TBZ. Thus, far, no study has compared TBZ and deuTBZ directly. A network meta-analysis of FIRST-HD and TETRA-HD studies showed that deuTBZ and TBZ had similar anti-chorea effect and safety profile, while patients receiving TBZ were more prone to experiencing depressive symptoms and somnolence (14). An indirect treatment comparison found a greater association between TBZ-use and neuropsychiatric adverse events, like akathisia and parkinsonism, compared to deuTBZ-use (15).

Clinical trial data is lacking on the management of non-motor symptoms. The clinical trial with buspirone to treat apathy had a negative result (16). Currently, dextromethorphan/quinidine and SRX46, a vasopressin 1A receptor antagonist, are being assessed for irritability (12), while psychiatric symptoms are treated based on expert consensus^1^. Selective serotonin reuptake inhibitors (SSRI) and serotonin-norepinephrine reuptake inhibitors (SNRI) are recommended for both depression and anxiety, while irritability is managed by sedative antidepressants, antipsychotics, or mood stabilizers. However, the treatment of apathy and cognitive symptoms in HD remains challenging. To improve cognition, two small clinical trials were not able to confirm the efficacy of cholinesterase inhibitors (17). A Phase 2 trial with SAGE-718 (NMDA receptor modulation) will start in the near future^2^ and a Phase1b open label trial is ongoing with nilotinib, to increase the dopamine level (NCT03764215).

### Cariprazine

Cariprazine (CAR) is a third-generation antipsychotic approved for the treatment of schizophrenia as well as for the depressive and manic and mixed episodes associated with bipolar I disorder in adult patients^3^. Furthermore, two studies had positive results for the adjunctive treatment of major depression disorder (MDD)^1^. Cariprazine is a dopamine D3 receptor preferring partial agonist at the D2/D3 receptors as well as at the serotonin 5-HT1A receptors, and acts as an antagonist at the 5-HT2B receptors (18). In fact, cariprazine's affinity to the D3 receptors is stronger than that of any other antipsychotics or even dopamine itself (19). Due to other antipsychotics' low affinity and dopamine's high affinity for the D3 receptors, antipsychotics (except for cariprazine) cannot occupy the D3 receptors in the presence of dopamine in the living brain (20). Therefore, only cariprazine is known to have the potential to dock to these receptors and exhibit the effects usually associated with D3 receptor blockade, which include improvements in negative, cognitive and depressive symptoms as well as in motivation and reward (21).

### Study Aims

This study aimed to explore the effects of 12-week cariprazine treatment on the mood and cognitive symptoms associated with Huntington's disease.

## Materials and Methods

### Patients

All patients had an abnormal expansion in the *HTT* gene (CAG >36) and were clinically diagnosed according to the diagnostic confidence level of the Unified Huntington's Disease Rating Scale (UHDRS). The diagnostic confidence level ranges from 0 (normal) to 4 (unequivocal extrapyramidal signs of HD, ≥99% confidence of the examiner).

The stage of the disease was identified by the Total Functional Capacity (TFC) of the UHDRS. Based on the TFC score, patients were classified into five stages that indicate levels of disease severity based on functional decline. Patients in Stage I had TFC scores of 11–13 (least severe); Stage II for scores 7–10; Stage III for scores 3–6; Stage IV for scores 1–2; and Stage V for a score of 0 (most severe).

All participants received the permission for off-label use of cariprazine issued by the Hungarian National Institute of Pharmacy and Nutrition. The study was conducted in accordance with the Declaration of Helsinki and all patients provided written informed consent.

### Study Design

This is a retrospective study aiming to evaluate the effect and safety of cariprazine in the treatment of non-motor (mood, behavioral, and cognitive) symptoms of Huntington's disease (Table 1). Efficacy and safety parameters were evaluated on week 8 and 12.

**Table 1:** Demographic and clinical measures of participants.

**Patient Id**	**Sex**	**Age**	**AOO**	**Repeats**	**TFC**	**Stage**	**Dose of CAR**	**Co-medication**	**Side effect**
P1	M	44	40	23/50	10	I	1.5 mg	Tetrabenazine 2 × 25 mg	None
P2	F	50	49	21/50	10	I	1.5 mg	Tetrabenazine 3 × 25 mg	None
P3	F	53	48	22/41	10	I	1.5 mg	Tetrabenazine 3 × 7.5 mg Paroxetin 1 × 20 mg	Akathisia
P4	F	56	54	23/42	7	II	1.5 mg	Alprazolam 3 × 0.5 mg	None
P5	F	50	45	21/48	12	I	3 mg	Glimepirid 1 × 4 mg	Akathisia
P6	F	38	31	19/47	10	I	1.5 mg	Tetrabenazine 4 × 25 mg Tiapridal 1 × 100 mg	None
P7	F	42	41	18/44	15	P	4.5 mg	None	None
P8	F	55	40	17/48	5	II	1.5 mg	Tetrabenazine 2 × 12.5 mg Clonazepam 3 × 0.5 mg Chlorprotixen 3 × 12.5 mg	None
P9	M	76	46	20/41	6	II	1.5 mg	Tetrabenazine 3 × 25 mg Alprazolam 1 × 0.25 mg	None
P10	F	45	41	23/48	6	II	1.5 mg	Tiapridal 3 × 100 mg Escitalopram 1 × 5 mg	None
P11	M	57	34	26/46	1	III	1.5 mg	Tetrabenazine 4 × 25 mg Sertraline 1 × 50 mg Clopazipne 1 × 25 mg	None
P12	F	44	40	15/48	10	I	1.5 mg	Tetrabenazine 3 × 12.5 mg Procyclidin 2 × 5 mg	Akathisia. Weight loss
P13	F	68	39	23/44	8	II	1.5 mg	Tetrabenazine 3 × 7.5 mg Paroxetin 1 × 20 mg	None
P14	F	45	40	16/40	5	II	1.5 mg	Tetrabenazine 3 × 50 mg Sertraline 1 × 50 mg	None
P15	M	44	42	21/48	12	I	1.5 mg	Tiapridal 3 × 100 mg	None
P16	F	37	36	20/48	12	I	1.5 mg	None	None

*Clinical stage is calculated on the basis of functional abilities (TFC, total functional capacity Score). Co-medication describes the concomitant pharmacotherapy given for the underlying condition. Side-effect column contains observations about adverse-events recorded to be attributed to administration of cariprazine. AOO, age of onset; CAR, cariprazine*.

Cariprazine was indicated if the patient had either mood symptoms (loss of motivation, apathy, anhedonia, depression) or cognitive alterations (executive dysfunction, planning difficulties, cognitive decline). The initial dose of CAR was 1.5 mg/day in the morning, which was increased to 3 mg/day if needed. Co-medications like tetrabenazine, benzodiazepines, antidepressants or antipsychotics were allowed if needed (Table 1). During the 12 week observational period of the study as new medication only procyclidine was introduced if akathisia appeared.

### Efficacy Evaluations

The study duration was 12 weeks. All efficacy parameters were evaluated at baseline, on week 8 and 12. Changes from baseline in mood and behavior were measured by the Beck Depression Inventory (BDI) and the Behavioral Assessment in the UHDRS scale. The BDI is a 21-item, self-report rating inventory that measures characteristic attitudes and symptoms of depression (22). The UHDRS scale was developed by the Huntington Study Group in 1996, updated in 1999 and its Cognitive and Behavioral Sections were clarified in 2005 (23).

Changes from baseline in cognitive performance was evaluated using the Addenbrooke Cognitive Examination (ACE) test and the Cognitive part of the UHDRS (computerized Stroop Test, Symbol Digit test, and Cognitive Verbal Fluency). The ACE consists of 19 activities in five cognitive domains: attention, memory, fluency, language and visuospatial processing (24, 25). The computerized Stroop Interference Test of the Vienna Test System (SCHUHFRIED GMBH Austria) was only performed at baseline, as it was highly challenging for the patients due to motor symptoms or cognitive impairment.

### Safety Evaluations

Safety assessments performed at baseline, 8 and week 12 included: body weight, vital signs, neurological examination, ECG, and routine laboratory testing along with assessments of motor functioning and adverse events.

### Statistical Analysis

Efficacy parameters were analyzed by mixed model for repeated measures (MMRM) separately for each parameter, with the terms of visit, baseline parameter value and their interaction, assuming unstructured covariance structure and using Kenward-Roger's approximation of the degrees of freedom. Least square (LS) means of the parameters (changes) by visits were estimated and compared between visits. Results are expressed as arithmetic means (+/- standard error) and statistics are related to the LS means (+/− standard error) of change from baseline (BL). If not otherwise stated, number of patients were 15. Because of the exploratory nature of the study, and since the changes might be correlated between the efficacy parameters, no adjustment for the possible increase of the type I error rate were applied, and differences were considered significant when *p* < 0.05.

## Results

### Patients

Our cohort consisted of four males and twelve female patients with a mean age of 48.13 years (± 26 yrs., SD 10.60) and mean disease duration of 3.78 years (± 6.22, SD 2.88). The average size of the CAG repeat expansion on the pathological allele was 46 (± 5, SD 3.28). One patient dropped out due to multiple events of non-compliance; hence, the presented efficacy analyses included data from 15 HD patients, while safety data included all 16 patients. One patient was in pre-symptomatic stage having 15 points in the TFC, eight were in Stage I, six in Stage II, and one in Stage IV (Table 1).

### Efficacy Outcomes

#### Mood and Behavioral Symptoms

**Figure 1:**
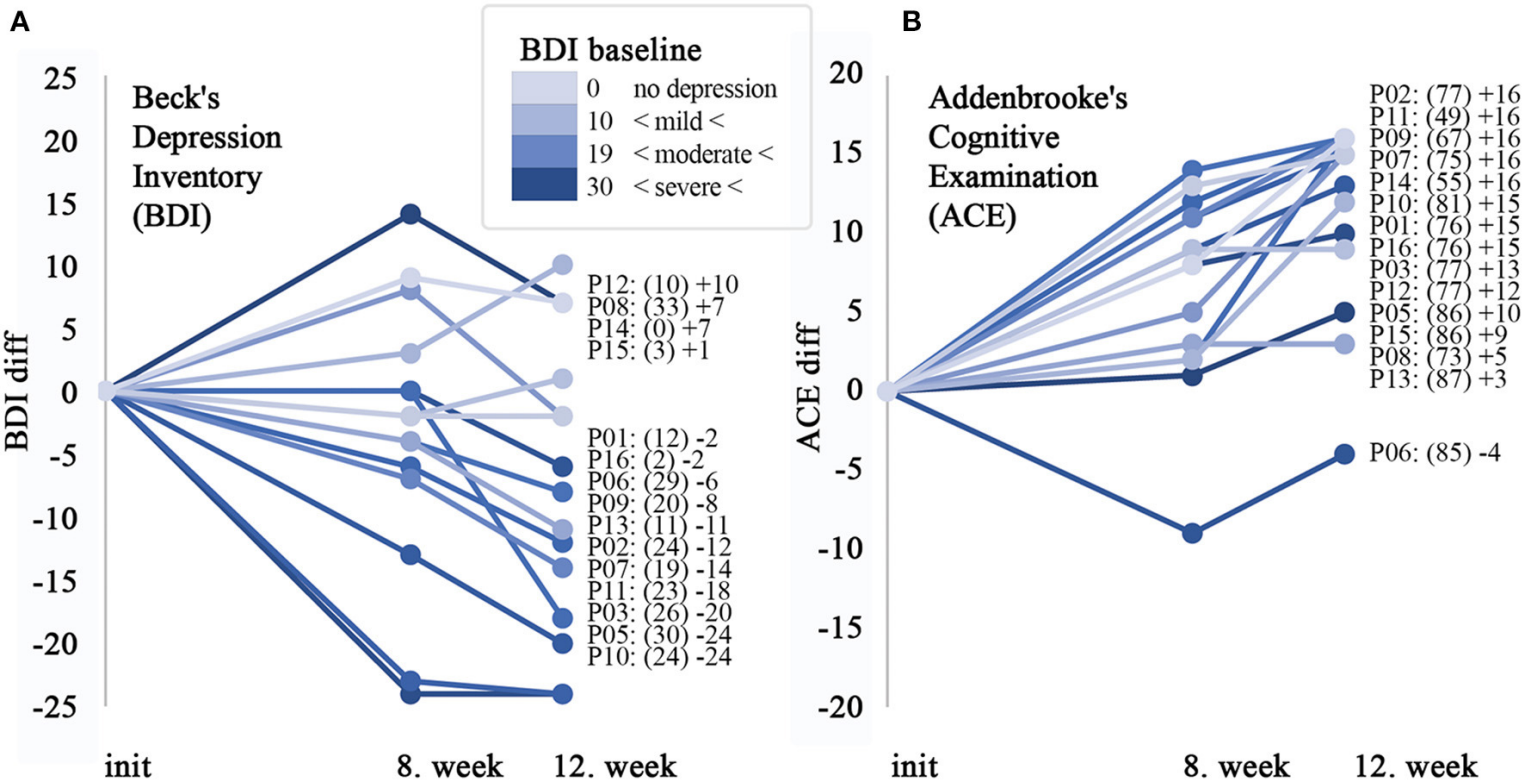
Significant improvement in cognitive performance, which can only partly be explained by the effect on depression. Alteration of the cognitive performance and depression status of participants with HD after starting administration of cariprazine. **(A)** Line plot shows the difference in individual points scored by Beck's Depression Inventory (0–63) compared to the baseline and 8 and 12th weeks, respectively. Next to the diagram, all of the participating patients are listed in the order of the largest difference in observed change over the observation period. The baseline points in brackets, followed by change at the 12th week is shown. **(B)** Line plot for Addenbrook's Cognitive Examination (ACE) questionnaire (0–100). On both diagrams, the color intensity of the lines is proportional with the severity on the depression scale in a similar way.

The severity of mood and apathy were evaluated by the Beck Depression Inventory and Behavioral Assessment from the UHDRS. The mean score of the BDI decreased from 17.7 + 10.7 (BL) to 10.0 + 10.7 (LS mean of change −7.7 +/−2.6 *p* < 0.0097) at week 12 (Figure 1A).

**Figure 2:**
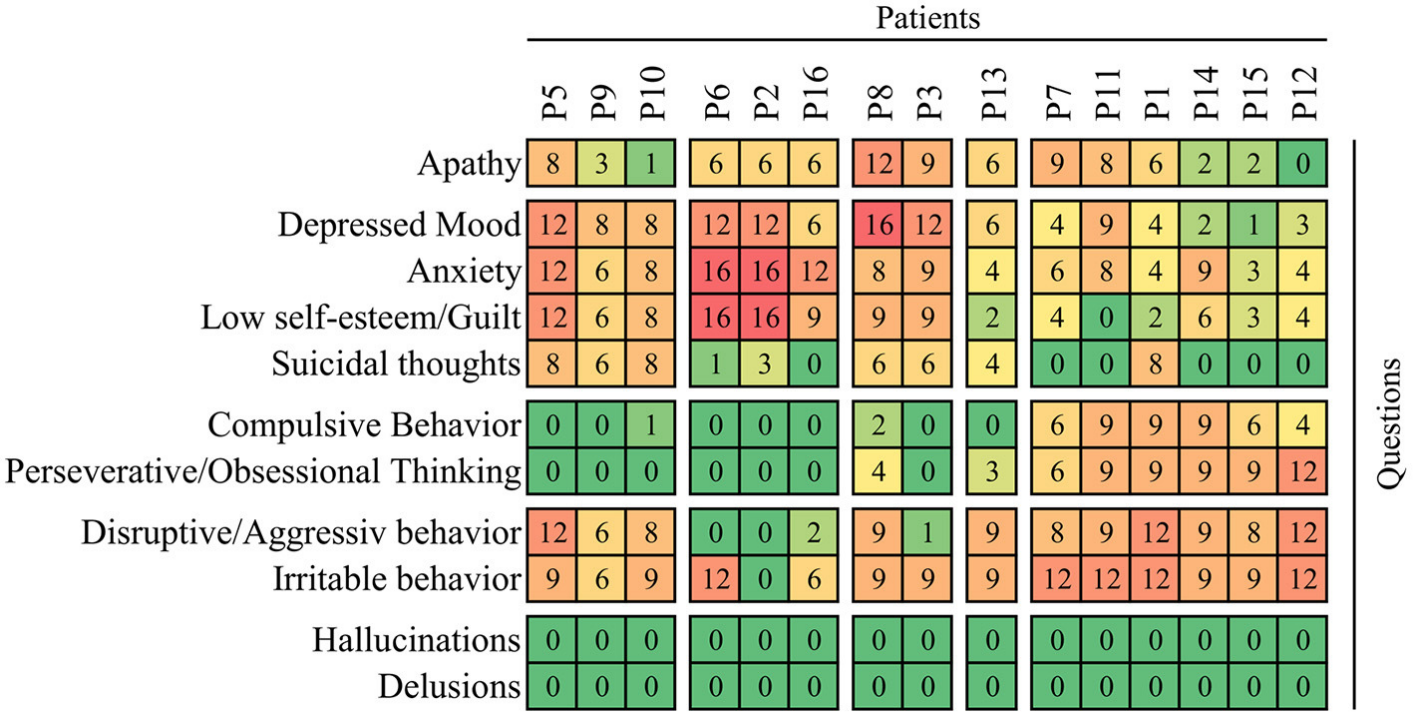
Individual profiles of behavioral impairment at baseline of observation before administration of cariprazine. The frequency x severity scores are shown on the heatmap with the maximum 16 points calculated as product of 4 (which means very frequently, most all the time on a 0–4 scale) and 4 (severe, causing a restriction of activities). The rows (questions/items) and columns (patients) are clustered on the basis of average correlation and separated in blocks according to first-order branches of dendrograms.

**Figure 3:**
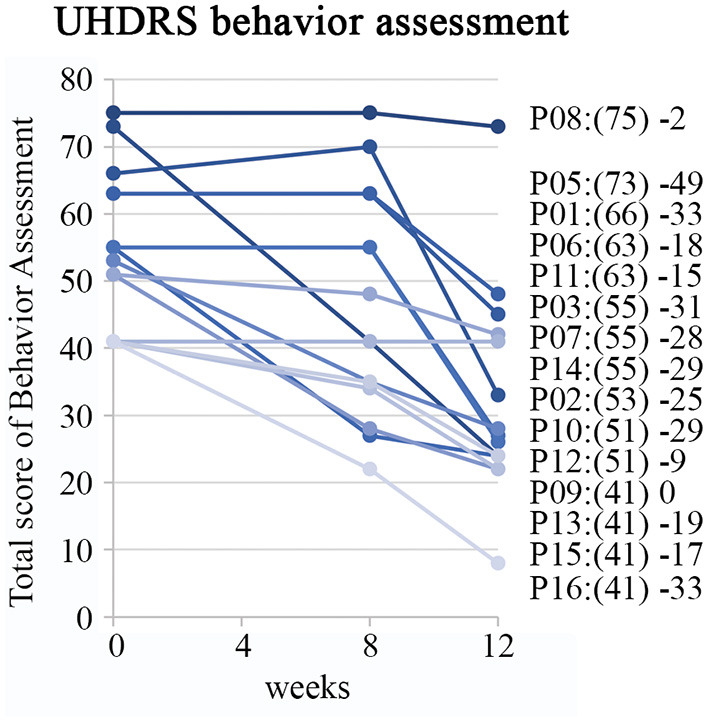
Improvement on behavior assessment measures of UHDRS. Individual patient profiles are shown with captions including baseline sum of frequency x severity scores in brackets and difference between baseline and assessed scores at the week 12.

Baseline scores of on the Behavioral Assessment in the UHDRS showed that irritability, anxiety, depression, low self-estimation, disruptive behavior and apathy were the most severe symptoms (Figure 2). The overall Behavioral Assessment score of the UHDRS decreased from 54.9 + 11.3 to 32.5 + 15.4, (LS mean change −22.5 3.4 *p* < 0.0001) after 12 weeks (Figure 3).

#### Cognitive Symptoms

Neuropsychological investigation detected the following changes regarding the cognitive functions: mean Addenbrooke Cognitive Examination total score increased from 75.1 + 11.0 (baseline) to 86.7 + 9.3 (week 12) (LS mean change 11.5+/−1.4 *p* < 0.0001, Figure 1B). The Cognitive Verbal fluency score of the Cognitive part of the UHDRS was 6.2 + 2.5 at the baseline, increased to 7.7 + 2.7 by week 12 (LS mean change 1.5+/−0.5, *p* = 0.0103). The mean baseline score of 9.2 + 6.9 on the Symbol Digit test increased to 12.3 + 8.9 by week 12 (LS mean change 3.1 +/−0.7, *p* = 0.0009, Table 2). The data of the baseline Stroop Interference tests are shown in the Supplementary Table.

**Table 2:** Differences during the observation period in cognitive and behavioral dimensions.

	**ACE**			**Symbol digit test**			**Verbal fluency**			**BDI**			**Behavioral assessment**		
	**BL**	**BL-W8**	**BL-W12**	**BL**	**BL-W8**	**BL-W12**	**BL**	**BL-W8**	**BL-W12**	**BL**	**BL-W8**	**BL-W12**	**BL**	**BL-W8**	**BL-W12**
P1	**76**	5	15	**20**	0	1	**6**	1	2	**12**	8	−2	**66**	4	−33
P2	**77**	12	16	**6**	0	3	**5**	1	3	**24**	−6	−12	**53**	−18	−25
P3	**77**	9	13	**6**	4	4	**7**	2	1	**26**	−13	−20	**55**	−28	−31
P5	**86**	8	10	**18**	5	9	**10**	2	4	**30**	−24	−24	**73**	−32	−49
P6	**85**	−9	−4	**8**	0	0	**8**	−2	−1	**29**	0	−6	**63**	0	−18
P7	**75**	11	16	**8**	0	2	**8**	0	1	**19**	−7	−14	**55**	0	−28
P8	**73**	1	5	**8**	0	−2	**8**	0	−3	**33**	14	7	**75**	0	−2
P9	**67**	14	16	**3**	0	2	**2**	1	3	**20**	−4	−8	**41**	0	0
P10	**81**	11	15	**4**	3	4	**6**	3	4	**24**	−23	−24	**51**	−23	−29
P11	**49**	2	16	**0**	0	1	**2**	0	1	**23**	0	−18	**63**	0	−15
P12	**77**	2	12	**6**	0	3	**7**	−1	1	**10**	3	10	**51**	−3	−9
P13	**87**	3	3	**20**	5	10	**6**	1	1	**11**	−4	−11	**41**	−7	−19
P14	**55**	8	16	**0**	0	2	**2**	0	2	**0**	9	7	**55**	0	−29
P15	**86**	9	9	**15**	2	2	**9**	2	0	**3**	−2	1	**41**	−6	−17
P16	**76**	13	15	**16**	4	6	**7**	2	3	**2**	−2	−2	**41**	−19	−33

*Individual baseline values (BL, in bold) are shown following by the differences between BL values at the baseline and week 8 or 12, respectively. Scores and in case of symbol digit test and verbal fluency correct answers within the specified time are indicated*.

### Safety Outcomes

The routine laboratory results (hematology and clinical chemistry) were within the normal range during the observation: serum glucose levels were slightly elevated in 3 patients at baseline (ranged between 6.3 and 7.1 mmol/l), however remained stable during the observation. Other laboratory parameters were within normal ranges. No significant changes were observed in vital signs, neurological examination, and ECG. Only a few patients reported experiencing side effects. Mild akathisia was the most frequent side effect, presenting in 2 of 16 patients (12.5%). No CAR related safety concerns arouse in motor functions.

## Discussion

To our knowledge, this is the first paper providing data on the efficacy of CAR in Huntington's disease. Next to studies proving cariprazine's efficacy in schizophrenia, mania and depression associated with bipolar I disorder recent large-scale studies showed efficacy in adjunctive MDD treatment as well^1^ (26, 27). Moreover, CAR is the only antipsychotic with proven superiority over another antipsychotic in the treatment of predominant negative symptoms, including anhedonia, avolition-apathy, and alogia (28). Furthermore, there are *post-hoc* analyses demonstrating improvement of cognitive dysfunction in different psychiatric disorders after CAR treatment (29–32). Positive observations have been reported further in the following indications: mitochondrial encephalomyopathy and lactic acidosis (MELAS syndrome) due to the mutation m.A3243G where the predominantly negative symptoms and cognitive dysfunction improved (33); substance use disorder (e.g., cocaine, alcohol, methamphetamine) (34, 35); obsessive-compulsive disorder as add-on therapy (36) and borderline personality disorder (37). Our study provides data for cariprazine's efficacy in non-motor symptoms of HD especially in loss of motivation, apathy, anhedonia, depression and cognitive symptoms.

In HD, *apathy* is one of the most common psychiatric symptoms, frequently occurring several years before the onset of motor symptoms. Studies suggest that between 11 and 64% of pre-symptomatic, and 47–76% of symptomatic HD patients have apathy (38–40). Another equally prevalent symptom in HD is depression. McAllister and colleagues (41) analyzed the prevalence, timing, and functional impact of psychiatric, cognitive, and motor abnormalities in HD in more than 6,000 individuals from the European Huntington's Disease Network^4^. They found that the most prevalent symptom after motor symptoms was depression, occurring in 64.5% of individuals with HD. Differentiation between depression and apathy would be important since their pharmaceutical and behavioral therapies may differ. However, the clinical differentiation is challenging since the definition of these entities is overlapping and not constant across diseases (42, 43). Apathy covers different aspects of a loss in motivation, which is commonly observed in many psychiatric and neurological disorders, including MDD, schizophrenia, Alzheimer's disease, Parkinson's disease, HD, ADHD, frontotemporal dementia, traumatic brain injury, post-traumatic stress disorders and stroke (38). The use of this terminology differs across patient groups, although it is now acknowledged that the underlying symptoms overlap greatly (38). In neurological disorders, loss of motivation is typically categorized as the syndrome of apathy, which itself is defined as diminished motivation for physical, cognitive and/or emotional activity (44). In psychiatry—with special reference to schizophrenia—loss of motivation corresponds to negative symptom domains such as avolition (lack of motivation, sense of purpose) and anhedonia (lack/loss of pleasure) (45) (loss of motivation = avolition, anhedonia). In HD many aspects can hinder the accurate diagnosis of apathy, like anergia, hopelessness and others on the negative affect and akinetic spectrum—a deeper understanding of their shared aspects is needed to better define and manage them in HD (40).

*Depression* can either be a disease (major depression or bipolar depression) with several ICD-10 (46) criteria needed to be met, or a symptom of decreased mood. Decreased mood can occur in various neuropsychiatric disorders, such as schizoaffective disorder, schizophrenia, bipolar disorder, MDD, stroke, Parkinson's disease, HD etc. In HD anxiety and irritability is frequently associated to the depression^4^.

In our study, apathy symptoms were measured by the Behavioral Assessment of the UHDRS, while depressive symptoms by the BDI. At baseline, irritability, anxiety, depression, low self-estimation, disruptive behavior and apathy dominated the clinical picture based on the Behavioral Assessment of the UHDRS. BDI detected moderate- to severe depression in 9 out of 15 patients. Both symptom domains improved significantly with CAR treatment. International guidelines for the treatment of Huntington's disease recommend the use of SSRIs or SNRIs for the treatment of either depression alone or depression combined with anxiety, suicidal ideation or impulsivity (47). However, there is a lack of evidence on specific antidepressant treatments in HD (48). A phase IIb multicentric, double-blind, placebo-controlled crossover trial with bupropion, a drug blocking the reuptake of dopamine and norepinephrine, failed to show any meaningful improvement targeting apathy in HD (16). In one case report, aripiprazole improved apathy induced by risperidone treatment (49). In our HD cohort, treatment with CAR resulted in significant improvements in both depression and apathy. This finding is crucial in the context of the large unmet need in the treatment of both apathy and depression in HD. Our study suggests that cariprazine might be a favorable therapeutic option for both symptoms.

The dopaminergic abnormalities are well-known in HD (for a comprehensive review, see Schwab et al.) (50). Altered DA signaling contributes not only to different component processes of reward, mainly mediating anticipatory phases, reinforcement processes and hedonic response (51, 52) but to cognitive manifestations of HD as well. The dysfunction of cognitive processing of emotion, similar to apathy, has been described in several CNS disorders such as depression (53), post-traumatic stress disorder (54) and progressive supranuclear palsy (55). In another dopamine associated disease, Parkinson's disease (PD) the cognitive deterioration was observed commonly in association with apathy (56, 57): apathetic PD patients had a significant decline in memory compared with non-apathetic patients (58). In Huntington's disease, cognitive deficit is also an important non-motor hallmark of the disease. Mild cognitive impairments as prefrontal symptoms are present prior to diagnosis in over half of the patients in early stages of HD (59). In many cases, similarly to psychiatric symptoms, cognitive deficits precede the onset of motor symptoms by years or even decades (41). A large multicentric study revealed that cognitive impairment is a very common feature besides depression, apathy and irritability (38). Patients with cognitive or behavioral symptoms had lower Total Functional Capacity (TFC) score of the UHDRS scale (41). In a longitudinal study of HD patients, half of those patients who were not affected by cognitive impairment at baseline experienced cognitive decline over time (60). There is a definite unmet need to improve the cognitive symptoms of HD especially in the early stage of the disease (61). In this study, treatment with cariprazine resulted in a significant improvement in the cognitive functions based on all test which was performed: the scores of the Addenbrooke Cognitive Examination, the Single Digit Modality and Verbal Fluency test significantly increased during the 12-week observational period. In our cohort, the cognitive scores improved in parallel with the BDI and UHDRS Behavioral Assessment Scores. It supports the hypothesis that by influencing the dopaminergic system, especially through the D3 receptors, the motivation and the cognitive functions can be improved (6).

Our present knowledge about the neurobiology of apathy, depression, and cognitive deficits suggests that there might be some shared mechanisms between these syndromes which are present in brain disorders associated with different etiology (38). Regarding apathy, the dysfunction of circuits connecting the PFC, basal ganglia and limbic system is believed to form the neurobiological basis (62, 63). The contributor effect of DA as a neurotransmitter in apathy besides its known involvement in the physiology of reward and hedonic response (51) is supported by the observations that in Parkinson's disease (64), and in patients with prefrontal or basal ganglia lesions, dopaminergic medication improved apathy (65), while ceasing dopaminergic medication after deep brain stimulation for PD increased apathy (64, 66).

In neurodegenerative disorders, neuropathological and neuroimaging studies revealed that apathy is strongly associated with lesion or functional impairment of the anterior cingulate cortex, ventromedial and dorsolateral PFC or ventral striatum and ventral tegmental area, as well as brain regions connected to these areas (67). Studies showed that compared to controls, there was a largely convergent network of brain regions with blunted activation during appetitive and decision-making tasks, as well as consummatory or learning phases of reward processing in patients with depression who have apathy (38). It is described that the same regions involved in the higher cognitive functions and the lesions of these areas are causing cognitive dysfunction (e.g., attention disturbances). Furthermore, in depression, activation is reduced in the above-mentioned regions, although contradictory results were reported in other studies (38). Martinez-Horta et al. (68) detected by PET that deterioration in apathy (as measured by the short version of the Problem Behaviors Assessment) significantly correlated with hypometabolism in the PFC, while the changes in depressive scores were correlated with hypometabolism in parietal-temporal regions in patients with pre-symptomatic Huntington's disease. In HD, decreases in the volume of the caudate, putamen, and the globus pallidus had the strongest correlations with clinical outcome measures for both motor and cognitive functions (69), however there is increasing evidence supporting the potential involvement of frontal lobe volume loss (68, 70).

Furthermore, the cognitive and mood symptoms (apathy and depression) might share common aetiological causes at neurotransmitter level: the dysfunction of the DA mesocortical pathway (71). In different psychiatric disorders, negative symptoms, like apathy, depression and cognitive impairment, have been associated with hypodopaminergic states in the prefrontal cortex (72). D3 partial agonist drugs, like CAR, increase dopamine levels in the PFC, normalizing the hypodopaminergic state. D3 receptors have been shown to play a distinct role in regulating excitability in layer 5 pyramidal cells in the PFC (73). Regional selective layer 5 pyramidal neuron degeneration correlates with clinical heterogeneity in HD symptom profiles (74). Also, the higher executive functions are linked to the PFC, which is partially damaged in HD. Given that the layer 5 pyramidal cells in the PFC contain D3 receptors, it can explain how HD causes impairments in motivation and how CAR is effective in restoring it.

There were some limitations to our study. Firstly, the present version of the Behavioral Assessment of the UHDRS has its limitations concerning the measurement of apathy, anhedonia and depression, since the questionnaire includes only one item intended to capture apathy and no item specifically focusing on anhedonia. Secondly, we only examined a small sample size and patient numbers were limited. Data should be confirmed by a study including a large sample size. Finally, longer follow-up time in a larger cohort is needed to validate our data.

In conclusion, our observations provide data about the positive effect of CAR on some psychiatric symptoms such as depressive mood, apathy and cognitive functions in patients with early stage of HD. We indicated that based on the neurobiological basis of these symptoms, CAR can improve the dopamine imbalance of the prefrontal cortex and thereby the symptoms themselves. This draws attention to a symptom-based transdiagnostic approach which supports the understanding of similar symptomatology in different neuropsychiatric disorders and helps identifying new indications of pharmaceutical compounds.

## Data Availability Statement

The original contributions presented in the study are included in the article/Supplementary Material, further inquiries can be directed to the corresponding author/s.

## Ethics Statement

The studies involving human participants were reviewed and approved by Regional and Institutional Research and Ethical Committee of Semmelweis University. The patients/participants provided their written informed consent to participate in this study.

## Author Contributions

MM and ZG: study design, patient management, neurological investigation, data interpretation, and writing the manuscript. VM: data collection, data interpretation, and writing manuscript. MF: neuropsychological testing. RC: data interpretation and writing the manuscript. KA: statistical analysis. BB: neurological investigation and writing the manuscript. All authors contributed to the article and approved the submitted version.

## Funding

This study was supported by the Hungarian National Brain Research Program KTIA_13_NAP-A-III/6 project and the FIKP program.

## Conflict of Interest

RC and KA are employees of Gedeon Richter Plc. The remaining authors declare that the research was conducted in the absence of any commercial or financial relationships that could be construed as a potential conflict of interest.

## Publisher's Note

All claims expressed in this article are solely those of the authors and do not necessarily represent those of their affiliated organizations, or those of the publisher, the editors and the reviewers. Any product that may be evaluated in this article, or claim that may be made by its manufacturer, is not guaranteed or endorsed by the publisher.

## Footnotes

^1^ https://www.gedeonrichter.com/en/news/211029

^2^ https://investor.sagerx.com/news-releases/news-releasedetails/sage-therapeutics-receives-fast-track-designation-sage-718

^3^ https://www.accessdata.fda.gov/drugsatfda_docs/label/2019/204370s006lbl.pdf

^4^ http://www.ehdn.org/
