# Supplementary material for: Improving Mood and Cognitive Symptoms in Huntington's Disease With Cariprazine Treatment
Source: Front Psychiatry. 2022 Feb 10;12:825532. doi: 10.3389/fpsyt.2021.825532 (PMC8866559; doi:10.3389/fpsyt.2021.825532)
Supplement: Supplementary file 1 [file Table_1.DOCX]

**Supplementary Table. Results of the Stroop test assessed by the software Stroop Interference Test of the Vienna Test System**

|  |  | **Interference tendency** | | | | | | **Detailed results - baseline** | | | | | | | | **Detailed results - interference conditions** | | | | | | | |  |
| --- | --- | --- | --- | --- | --- | --- | --- | --- | --- | --- | --- | --- | --- | --- | --- | --- | --- | --- | --- | --- | --- | --- | --- | --- |
|  | Edu level | Reading interference tendency | | | Naming interference tendency | | | MRT - Reading | | | MRT - Naming | | | NIR - Reading | NIR - Naming | MRT - Reading | | | MRT - Naming | | | NIR - Reading | NIR - Naming | Working time |
|  |  | **raw** | **PR** | **T** | **raw** | **PR** | **T** | **raw** | **PR** | **T** | **raw** | **PR** | **T** | **raw** | **raw** | **raw** | **PR** | **T** | **raw** | **PR** | **T** | **raw** | **raw** | **raw** |
| P1 | 5 | 0.32 | 6 | 34 | 0.14 | 38 | 47 | 0.75 | 45 | 49 | 0.72 | 37 | 47 | 2 | 5 | 1.07 | 15 | 40 | 0.86 | 40 | 47 | 7 | 2 | 7:48 |
| P2 | 4 | 0.51 | 1 | 27 | 0.69 | 1 | 27 | 1.17 | 2 | 29 | 1.09 | 2 | 29 | 0 | 0 | 1.68 | 1 | 27 | 1.78 | 1 | 27 | 0 | 1 | 13:05 |
| P3 | 4 | 1.72 | 0 | 20 | 0.23 | 17 | 40 | 1.43 | 0 | 20 | 1.41 | 0 | 20 | 0 | 0 | 3.14 | 0 | 20 | 1.64 | 2 | 29 | 3 | 1 | 18:53 |
| P4 |  |  |  |  |  |  |  |  |  |  |  |  |  |  |  |  |  |  |  |  |  |  |  |  |
| P5 | 4 | 0.43 | 2 | 29 | 0.5 | 2 | 29 | 0.86 | 16 | 40 | 0.88 | 10 | 37 | 0 | 0 | 1.3 | 4 | 32 | 1.38 | 3 | 31 | 6 | 4 | 10:50 |
| P6 | 2 | # | # | # | 0.09 | 65 | 54 | 2.26 | 0 | 20 | 1.96 | 0 | 20 | 0 | 3 | 2.24 | 0 | 20 | 2.05 | 0 | 20 | 5 | 3 | 19:39 |
| P7 | 5 | 0.29 | 9 | 37 | 0.96 | 0 | 20 | 1.11 | 2 | 29 | 1.05 | 2 | 29 | 1 | 3 | 1.4 | 2 | 29 | 2.02 | 0 | 20 | 5 | 2 | 13:58 |
| P8 | N/D | # | # | # | # | # | # | # | # | # | # | # | # | # | # | # | # | # | # | # | # | # | # | # |
| P9 | N/D | # | # | # | # | # | # | # | # | # | # | # | # | # | # | # | # | # | # | # | # | # | # | # |
| P10 | 4 | 0.33 | 6 | 34 | 0.63 | 1 | 27 | 1.62 | 0 | 20 | 1.48 | 0 | 20 | 4 | 5 | 1.95 | 0 | 20 | 2.12 | 0 | 20 | 5 | 7 | 16:26 |
| P11 | N/D | # | # | # | # | # | # | # | # | # | # | # | # | # | # | # | # | # | # | # | # | # | # | # |
| P12 | 4 | 0.21 | 20 | 42 | 0.13 | 42 | 48 | 1.15 | 2 | 29 | 1.2 | 1 | 27 | 1 | 2 | 1.36 | 3 | 31 | 1.32 | 4 | 32 | 4 | 8 | 11:56 |
| P13 | 4 | 0.19 | 27 | 44 | 0.28 | 12 | 38 | 1.34 | 0 | 20 | 1.29 | 0 | 20 | 3 | 0 | 1.54 | 1 | 27 | 1.57 | 2 | 29 | 6 | 15 | 13:15 |
| P14 | N/D | # | # | # | # | # | # | # | # | # | # | # | # | # | # | # | # | # | # | # | # | # | # | # |
| P15 | 4 | 0.72 | 0 | 20 | 0.14 | 35 | 46 | 1.01 | 4 | 32 | 0.91 | 7 | 35 | 0 | 0 | 1.73 | 1 | 27 | 1.05 | 12 | 38 | 6 | 0 | 10:57 |
| P16 | 4 | 0.05 | 88 | 62 | 0.1 | 54 | 51 | 0.88 | 14 | 39 | 0.79 | 25 | 43 | 5 | 4 | 0.92 | 36 | 46 | 0.89 | 36 | 46 | 6 | 3 | 7:56 |

# Missing data due to the failure of the testing

Edu level Education level

MRT Median for reaction times (sec.)

NIR Number of incorrect reactions
